# A Histone Code Functionally Linked to Replicative Senescence

**DOI:** 10.1111/acel.70343

**Published:** 2026-01-02

**Authors:** Thomas Suter, Meyer J. Friedman, Cagdas Tazearslan, Amir Gamliel, Daria Merkurjev, Kenneth Ohgi, Zhongjun Zhou, Michael G.Rosenfeld, Yousin Suh

**Affiliations:** ^1^ Cellular and Molecular Medicine, Department of Medicine University of California San Diego La Jolla California USA; ^2^ Department of Genetics Albert Einstein College of Medicine Bronx New York USA; ^3^ School of Biomedical Sciences, Li Ka Shing Faculty of Medicine The University of Hong Kong Hong Kong China; ^4^ Shenzhen Institute of Innovation and Research The University of Hong Kong Nanshan Shenzhen China; ^5^ Department of Obstetrics and Gynecology Columbia University Irving Medical Center New York USA; ^6^ Department of Genetics and Development Columbia University Irving Medical Center New York USA

**Keywords:** aging, gene length, H3K79me^3^, H4R3me^(2as)^, H4R3me^(2s)^, histone code, replicative senescence, transcription

## Abstract

Cell states and biological processes are defined by their epigenetic profiles, distinctive composites of DNA‐ and histone‐based chromatin components. However, the specific histone posttranslational modifications that distinguish cellular senescence and the impact of their distribution on transcription, especially with regard to gene length, have not been fully elucidated. Here, we show that promoter loss of symmetric dimethylated H4R3 (H4R3me^(2s)^) and spreading of trimethylated H3K79 (H3K79me^3^) across gene bodies are functional features of replicative senescence associated with gene upregulation. We report that highly upregulated genes in replicative senescence exhibit enrichment of H3K79me^3^ and, in contrast to the characteristic trend of aging cells and tissues, are substantially longer than those that are significantly downregulated. Furthermore, by assessing all expressed genes, we demonstrate that gene body accumulation of H3K79me^3^ during the transition to replicative senescence constitutes a broader phenomenon that is positively correlated with gene length and expression level genome‐wide. Consistently, pharmacological inhibition of H3K79me^3^ deposition attenuates gene upregulation in replicative senescence. We also document a striking increase in the levels of H3K79me^3^ as well as a robust H4R3me^(2s)^ to asymmetric dimethylated H4R3 (H4R3me^(2as)^) epigenetic switch that manifest globally in late‐passage cells, suggesting that these histone modifications might represent novel molecular biomarkers of replicative senescence. Finally, we implicate the associated epigenetic regulators, including DOT1L, PRMT1, PRMT5, and JMJD6, as modifiers of cellular lifespan, potentially disclosing unappreciated therapeutic targets for interventions in normal and pathological aging. Collectively, our findings provide novel insights into the histone code that mediates altered transcriptional regulation in replicative senescence.

## Graphical abstract

In replicatively‐senescent fibroblasts, promoter loss of H4R3me^(2s)^ and gene body spreading of H3K79me^3^ facilitate transcriptional upregulation, which preferentially occurs at long genes. These cells also feature striking shifts in the global levels of H4R3me^(2s)^, H4R3me^(2as)^, and H3K79me^3^. Suppression of these epigenetic changes attenuates replicative senescence‐associated transcriptional alterations and extends cellular lifespan, demonstrating their functional significance.

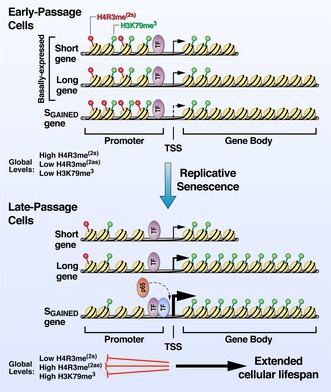

Epigenetic alterations are considered a hallmark of cellular senescence, facilitating the transcriptional changes that underlie characteristic senescent features, including cell cycle arrest and the senescence‐associated secretory phenotype (SASP) (López‐Otín et al. 2023). Indeed, changes in 3D nuclear architecture, DNA methylation patterns, overall histone content, histone variant incorporation, and covalent modifications of canonical histones, primarily involving acetylation or methylation of specific lysine residues in H3 and H4, have previously been documented among others in senescent cells (Dasgupta et al. 2024). While altered enrichment of enhancer (H3K27Ac and H3K4me^1/2^)– as well as promoter (H3K4me^3^)–defining histone marks has been exploited to identify cis regulatory elements (CREs) and constituent transcription factors (TFs) that collectively drive senescent transcriptional programs (Martínez‐Zamudio et al. 2020; Dasgupta et al. 2024; Suter et al. 2025), the contribution of other histone post‐translational modifications to transcriptional regulation in the different types of cellular senescence has not been completely resolved.

To assess senescence‐associated transcriptional changes, we further analyzed our RNA‐seq and GRO‐seq data from early‐passage quiescent (EPQ) and senescent BJ fibroblasts (Figures 1a and S1a), which had identified 639 upregulated (S_GAINED_) and 598 downregulated (S_LOST_) genes in replicative senescence (Suter et al. 2025). These datasets contained canonical senescence genes, such as *CDKN1A*, *IGF1*, and several encoding TGF‐β family ligands in the former and *BUB1B* as well as *LMNB1* in the latter (Table S1). Consistently, enriched GO terms for the S_GAINED_ genes included activation of secretion, growth regulation, and p53 signaling (Figure 1b), while S_LOST_ genes were functionally associated with cell cycle and DNA replication (Figure S1b). Unexpectedly, given the documented inverse correlation between the length of genes and their transcriptional output in aged cells and tissues (Vermeij et al. 2016; Stoeger et al. 2022; Ibañez‐Solé et al. 2023), a phenomenon known as gene length‐dependent transcription decline (GLTD) (Soheili‐Nezhad et al. 2024), S_GAINED_ genes were markedly longer than those of the S_LOST_ or unchanged gene sets, which did not differ significantly in length (Figures 1c and S1c). Analysis of the GRO‐seq data also suggested substantial promoter‐proximal RNA polymerase II (Pol II) pausing regulation in replicative senescence (Figure S1d,e).

**FIGURE 1:**
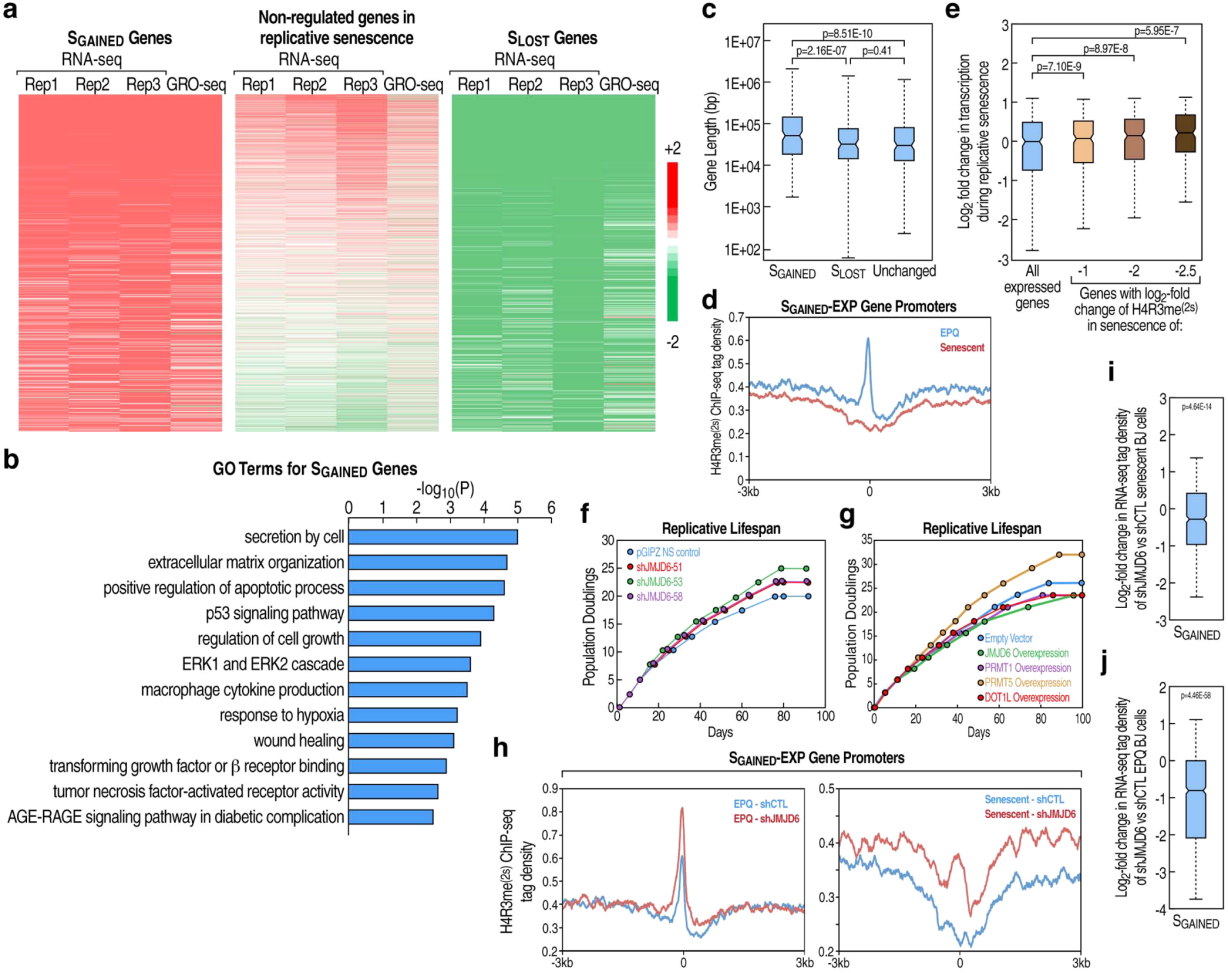
Loss of promoter H4R3me^(2s)^ at transcriptionally‐upregulated genes is functionally associated with replicative senescence. (a) Heatmap of transcriptional changes observed in replicative senescence for S_GAINED_, S_LOST_, and non‐regulated gene sets. Increase (red) or decrease (green) in normalized tags over gene exons (RNA‐seq) or bodies (GRO‐seq) in senescent BJ fibroblasts relative to EPQ cells is shown. (b) Selected GO‐terms from Metascape analysis (Zhou et al. 2019) of 639 S_GAINED_ genes. Negative log_10_P values for each term are plotted. (c) Box plot showing the distribution of gene lengths for S_GAINED_, S_LOST_, and unchanged gene sets in replicative senescence. *p* values determined by two‐sided Mann–Whitney *U* tests. See methods for additional details regarding box plots and significance testing. (d) Meta‐analysis distribution of normalized H4R3me^(2s)^ native ChIP‐seq tag density in senescent or EPQ BJ fibroblasts, centered on the TSSs of S_GAINED_‐Expanded (S_GAINED_‐EXP) genes, an expanded set of S_GAINED_ genes (see methods). (e) Box plot of log_2_ fold change in transcription during senescence of either all genes expressed in BJ fibroblasts or only genes with the indicated senescence‐associated decrease in H4R3me^(2s)^ in a −300 to +200 bp window relative to their TSSs. *p* values determined by two‐tailed *z*‐tests. (f) Population doublings over time of BJ fibroblasts after shRNA‐mediated stable knockdown of JMJD6 versus shRNA non‐silencing (NS) control vector. JMJD6‐51, −53, and −58 represent distinct shRNA constructs. (g) Population doublings over time of BJ fibroblasts expressing doxycycline‐inducible HA‐DOT1L, ‐JMJD6, ‐PRMT1, or ‐PRMT5 versus empty control vector in the continuous presence of 20 nM doxycycline. (h) Meta‐analysis of normalized H4R3me^(2s)^ native ChIP‐seq tag density distribution, centered on the TSSs of S_GAINED_‐EXP genes (as in (d)), for EPQ (left) and senescent (right) BJ fibroblasts stably expressing control or JMJD6 shRNA. (i) Box plot of log_2_ fold change in RNA‐seq tag density for the S_GAINED_ gene set in shJMJD6 stable knockdown versus shRNA non‐silencing control senescent BJ fibroblasts. *p* value determined by two‐tailed *z*‐test. (j) Box plot of log_2_ fold change in RNA‐seq tag density for the S_GAINED_ gene set in shJMJD6 stable knockdown versus shRNA non‐silencing control EPQ BJ fibroblasts. *p* value determined by two‐tailed *z*‐test.

Our earlier work linked the removal of enhancer‐localized H4R3me^(2s)^ with increased Pol II pause release at the promoters of a cohort of physically‐associated genes (Liu et al. 2013), while other reports have connected H4R3me^(2s)^ promoter occupancy with transcriptional repression and that of its asymmetric counterpart, H4R3me^(2as)^, with transcriptional activation (Baldwin et al. 2014; Kim et al. 2016; Xu et al. 2010). H4R3me^(2s)^ ChIP‐seq analysis of genes induced during senescence revealed a striking transcription start site (TSS) enrichment in EPQ BJ fibroblasts that disappeared entirely in senescent cells (Figure 1d). Genes with the greatest reduction in TSS H4R3me^(2s)^ were most induced during senescence (Figure 1e), indicating that removal of this mark is functionally relevant in transcription and consistent with its documented association with transcriptional repression (Baldwin et al. 2014; Kim et al. 2016; Xu et al. 2010; Li et al. 2018). GO functional analysis of genes displaying robust promoter loss of H4R3me^(2s)^ in replicative senescence demonstrated enrichment of terms related to cell cycle regulation but also autophagy and translation (Figure S1f), possibly connecting this altered histone modification with the selective contributions of the latter processes to the SASP (Kwon et al. 2017; Payea et al. 2021). In addition, H4R3me^(2s)^ promoter depletion during senescence was associated with occupancy of the NF‐κB subunit p65, which progressively increased at S_GAINED_ gene promoters (Figure S1g), consistent with its well‐established role in driving inflammatory SASP gene expression (Kolesnichenko et al. 2021; Saul et al. 2022; Suter et al. 2025). While the change in H4R3me^(2s)^ levels was more pronounced at p65‐binding promoters, it was not specific to this subset (Figure S1h). Thus, the promoter residency of H4R3me^(2s)^ may modulate the recruitment of various activating TFs in replicative senescence.

Notably, H4R3 methylation status appeared to impact the onset of proliferative arrest in cultured fibroblasts. Knockdown of JMJD6, an eraser of H4R3me^(2s)^, attenuated senescence‐associated upregulation of *CDKN1A* (Figure S1i), which encodes the cyclin‐dependent kinase inhibitor p21, and increased the replicative lifespan of BJ cells (Figures 1f and S1j). Overexpression of PRMT5, a writer of H4R3me^(2s)^, also delayed the discontinuation of cellular proliferation (Figure 1g). In contrast, overexpression of either JMJD6 or the primary writer of H4R3me^(2as)^, PRMT1, accelerated the induction of replicative senescence (Figure 1g). Genes with the greatest loss of H4R3me^(2s)^ during replicative senescence were downregulated upon JMJD6 knockdown in senescent cells (Figure S1k). As expected, knockdown of JMJD6 caused an increase in the H4R3me^(2s)^ signal at the promoters of senescence‐induced genes in replicatively senescent and EPQ cells (Figure 1h); however, it is unclear if the effect may have been limited by residual JMJD6 or, alternatively, the involvement of an additional arginine demethylase (Zhang et al. 2019). Nevertheless, S_GAINED_ gene expression was significantly reduced by shJMJD6 in both cellular contexts (Figure 1i,j). Collectively, these results demonstrate a role for JMJD6 in H4R3me^(2s)^ removal and S_GAINED_ gene de‐repression in replicative senescence.

We also evaluated the potential involvement of H3K79me^3^ in replicative senescence, as this histone mark has recently been linked to increased transcription efficiency by reducing Pol II pausing duration (Huynh et al. 2023). The non‐SET domain lysine methyltransferase DOT1L acts in a non‐processive manner to catalyze mono‐, di‐, and tri‐methylation of H3K79 (Min et al. 2003; Shahbazian et al. 2005). H3K79me^3^ generally shows enrichment at the 5′ end of expressed genes that extends, in a diminishing fashion, into the gene body (Wood et al. 2018), and the modification is strongly correlated with active transcription (Jonkers et al. 2014; Steger et al. 2008; Veloso et al. 2014; Wang et al. 2008). ChIP‐seq data demonstrated a dramatic enrichment of H3K79me^3^ on many S_GAINED_ genes, as exemplified by the TNFRSF10 gene cluster (Figure S2a). Furthermore, assessment of all expressed genes revealed increased H3K79me^3^ decoration of gene bodies genome‐wide in replicative senescence, which was distinct from its promoter‐proximal enrichment that characterized EPQ cells (Figures 2a,b and S2a). A similar distribution was observed for H3K79me^2^ (Figure S2a), albeit with less pronounced 5′ genic accrual in early‐passage cells, as evinced by metagene analysis of ChIP‐seq data (Figure 2c). In contrast, the presence of K120‐monoubiquitinated H2B (H2Bub), which is a prerequisite for the processive methylation of monomethylated H3K79 (Shahbazian et al. 2005; Yao et al. 2015), was unchanged genome‐wide during replicative senescence (Figures 2d and S2a). Consistent with its redistribution, we detected a greater increase in H3K79me^3^ on long gene bodies in replicatively senescent cells (Figures 2e and S2b).

**FIGURE 2:**
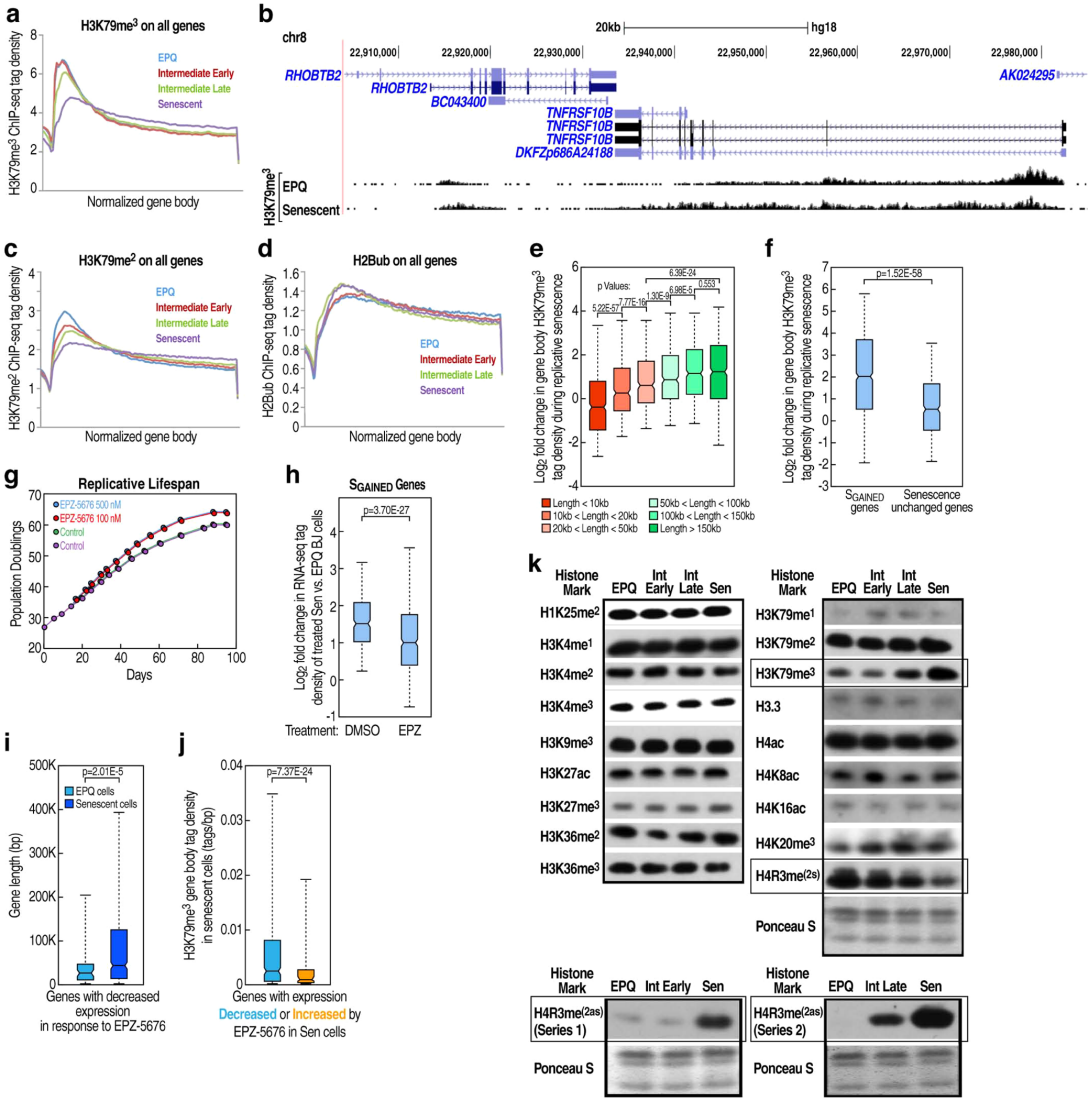
Gene body spreading of H3K79me^3^ at transcriptionally‐upregulated genes is functionally associated with replicative senescence. (a) Meta‐analysis of H3K79me^3^ levels across normalized gene bodies of all expressed Refseq genes at indicated cell stages. (b) Representative UCSC browser snapshot of H3K79me^3^ distribution in EPQ and senescent BJ fibroblasts. (c) Meta‐analysis of H3K79me^2^ levels across normalized gene bodies of all expressed Refseq genes at indicated cell stages. (d) Meta‐analysis of H2BK120Ub (H2Bub) levels across normalized gene bodies of all expressed Refseq genes at indicated cell stages. (e) Box plot of log_2_ fold change in normalized gene body H3K79me^3^ tag density during replicative senescence for all BJ fibroblast‐expressed genes in different gene‐length categories. (f) Box plot of log_2_ fold change in normalized gene body H3K79me^3^ tag density during replicative senescence comparing S_GAINED_ genes with transcriptionally‐unchanged genes (i.e., expressed genes classified as neither S_GAINED_ nor S_LOST_). (g) Population doublings over time of BJ fibroblasts treated with EPZ‐5676 or DMSO vehicle control. (h) Box plot of log_2_ fold change in RNA‐seq tag density (ratio of normalized tag counts) for S_GAINED_ genes in senescent (PD 64) versus EPQ (PD 30) BJ fibroblasts following DMSO or EPZ‐5676 treatment. (i) Box plot of gene length (bp) for downregulated genes (< −1 log_2_ fold change in normalized RNA‐seq tag density) in either EPQ or senescent cells after prolonged EPZ‐5676 treatment. (j) Box plot of normalized H3K79me^3^ gene body tag density in untreated senescent BJ fibroblasts for gene sets with decreased (< −1 log_2_ fold change) or increased (> 1 log_2_ fold change) expression, based on normalized RNA‐seq tag density, in EPZ‐5676‐treated senescent cells. (k) Western blot analysis of various histone modifications in BJ fibroblasts at different cell stages. Samples were normalized to total histone levels, as confirmed by Ponceau S staining (see methods). EPQ, Intermediate (Int) Early, and Senescent (Sen) histone extracts correspond to PD 19, PD 30, and PD 78, respectively. Int Late represents PD 46 in the upper panels and PD 50 in the lower panel (series 2). For box plots in (e), (f), (h), (i), and (j), *p* values were determined by two‐tailed *z*‐tests.

H3K79me^3^ spreading across gene bodies correlated with elevated transcription, as S_GAINED_ genes, representing the most robustly upregulated subset, showed a greater increase in H3K79me^3^ relative to transcriptionally unchanged genes in replicatively senescent fibroblasts (Figure 2f). Globally, long genes displayed significantly higher transcription (Figure S2c) and lower rates of pausing (Figure S2d) in replicative senescence. Furthermore, culturing BJ fibroblasts in media containing EPZ‐5676, a highly specific and potent DOT1L inhibitor (Figure S2e), increased maximum population doublings (Figure 2g), whereas sustained overexpression of DOT1L resulted in earlier onset of proliferative arrest (Figures 1g and S2f). EPZ‐5676 treatment attenuated senescence‐associated upregulation of S_GAINED_ genes (Figure 2h), and its effects were enhanced in combination with rapamycin (Figure S2g), which has a well‐established capacity to extend the lifespan of cultured cells and model organisms (Johnson et al. 2013). These drugs, individually and in combination, similarly impacted changes in S_LOST_ genes (Figure S2h). In accord with H3K79me^3^ being conducive to transcription, for both EPQ and senescent cells treated with EPZ‐5676, genes that were transcriptionally repressed by the drug showed a greater decrease in H3K79me^3^ relative to induced genes (Figure S2i,j). In addition, EPZ‐5676‐repressed genes in senescent cells were considerably longer than those repressed by the inhibitor in EPQ cells (Figure 2i), consistent with the expansion of H3K79me^3^ over long genes in replicative senescence. While genes repressed by EPZ‐5676 were similar in length to inhibitor‐induced genes in senescent cells (Figure S2k), the former displayed considerably higher levels of H3K79me^3^ (Figure 2j). Furthermore, compared to the drug‐induced subset, EPZ‐5676‐repression‐sensitive genes featured a greater increase in H3K79me^3^ during replicative senescence (Figure S2l), while exhibiting higher transcription (Figure S2m) and H3K79me^3^ promoter occupancy (Figure S2n) in EPQ cells. Collectively, these results indicate that H3K79me^3^ contributes to the robust induction of many S_GAINED_ genes as well as a more modest, length‐dependent upregulation of basally‐expressed genes in replicative senescence. It was recently reported that elevated H3K79me^3^ is only discernable at a specific SASP gene locus in oncogene‐induced senescence and that DOT1L does not impact proliferation arrest therein (Leon et al. 2021). Thus, the effects of H3K79me^3^ and its writer in cellular senescence may be context dependent.

To confirm that genes altered in replicative senescence are distinct from those subject to GLTD, we overlapped the S_GAINED_ and S_LOST_ genes with a published compilation of human age‐related differentially expressed genes (DEGs) (Jia et al. 2018) based on bulk RNA‐seq data from multiple tissues generated by the Genotype‐Tissue Expression (GTEx) project (Lonsdale et al. 2013) (Figure S3a). The gene length distributions of the upregulated (UAG) and downregulated age‐associated gene (DAG) lists were consistent with GLTD bias against long gene expression (Figure S3b), and removal of all overlapping S_GAINED_ and S_LOST_ genes had almost no effect on the gene length disparity of UAGs versus DAGs (Figure S3c). Thus, the senescent gene cohort is separable from those transcription units undergoing GLTD in aging and may constitute an important exception to the phenomenon. We also performed enrichment analysis of the S_GAINED_ and S_LOST_ gene sets in GTEx age‐related, tissue‐stratified DEGs using the voyAGEr platform (Schneider et al. 2024), which revealed significant over‐representation of both groups in multiple tissues from middle‐aged and/or older individuals. This enrichment was particularly robust in several tissues whose aging‐linked DEGs were previously reported either to defy the general GLTD trend of long gene downregulation or to show no significant difference with regard to gene length in GTEx transcriptomic data (Stoeger et al. 2022) (Figure S3d; Table S2). These observations suggest the possibility that sufficient accumulation of cells with a senescent or senescent‐like transcriptional profile may help to explain, at least in part, the documented lack of uniformity in the GLTD phenotype (Stoeger et al. 2022).

Our results support a model in which H3K79me^3^, after initially accumulating at gene promoters in early‐passage cells, spreads into gene bodies as cell cycle progression slows during the onset of replicative senescence to facilitate increased transcription of basally‐expressed genes in a length‐dependent fashion. H3K79me^3^ gene body enrichment also supports the robust induction of senescence‐defining genes (i.e., S_GAINED_ genes). Thus, H3K79me^3^ spreading may constitute an unappreciated molecular mechanism by which transcriptional upregulation of replicative senescence‐associated genes is achieved, despite their typically longer length. Redistribution of H3K79me^3^ might also have indirect effects that enable elevated gene expression, potentially modulating architectural features within a given topologically associating domain (TAD) to increase overall transcriptional potential therein (Dasgupta et al. 2024; Suter et al. 2025). H3K79me^3^ spreading across gene bodies occurs in conjunction with JMJD6‐mediated loss of promoter H4R3me^(2s)^ that may be conducive to the binding of p65 and other activating TFs. Collectively, these chromatin events, which are likely coordinated with enhancer‐driven programs (Martínez‐Zamudio et al. 2020; Friedman et al. 2024; Suter et al. 2025), contribute to the altered transcriptional landscape in replicative senescence.

Notably, we detected marked changes in the global levels of H3K79me^3^, H4R3me^(2s)^, and H4R3me^(2as)^ in late‐passage BJ fibroblasts (Figure 2k), raising the prospect that these particular histone modifications could serve as novel molecular biomarkers of replicative senescence. Their association with tissue aging, given its heterogeneity (Rando and Wyss‐Coray 2021), will require systematic evaluation in vivo. In addition, the implicated epigenetic regulatory enzymes warrant further investigation, especially with regard to the detailed mechanism(s) by which they impact replicative lifespan, as they may constitute innovative targets for anti‐aging interventions seeking to mitigate the deleterious effects of cellular senescence (Zhang et al. 2022; López‐Otín et al. 2023).

## Author Contributions

Y.S., M.G.R., T.S., and C.T. conceived the project. T.S. generated most of the constructs, established modified cell lines, and performed functional genomics assays. C.T. carried out the long‐term cultures and PD curves. T.S., A.G., and D.M. performed the bioinformatic analyses. Sample preparations for deep sequencing were done by K.O. and T.S. Preliminary in vivo data were provided by Z.Z. Revision experiments/analyses were performed by M.J.F. T.S., M.J.F., M.G.R., and Y.S. wrote and edited the manuscript, with input from C.T.

## Funding

This work was supported by NIH grants to Y.S. and M.G.R. (R01AG069750, R01DK127778, R01AG057706, and 5R01AG06152).

## Conflicts of Interest

The authors declare no conflicts of interest.

## Supporting information


**Appendix S1:** Methods and Supplementary Figures.
**Figure S1:** Further characterization of S_GAINED_ and S_LOST_ genes and the functional link of H4R3me^(^^2s^^)^ promoter depletion to replicative senescence. (a) Box plot of fold change in expression of S_GAINED_ and S_LOST_ genes during BJ fibroblast replicative senescence. Values correspond to average fold change in senescent versus EPQ cells based on RNA‐seq triplicates and a single GRO‐seq assay. *p* values determined by two‐tailed *z*‐tests. (b) Selected GO‐terms from Metascape analysis of 598 S_LOST_ genes. Negative log_10_ *p* values for each term are plotted. (c) Box plot showing the distribution of gene lengths for S_GAINED_, S_LOST_, and unchanged gene sets in replicative senescence, as in Figure 1c, but including only protein‐coding genes. *p* values determined by two‐sided Mann–Whitney *U* tests. (d) Pausing index (PI) curves for senescent up‐ and down‐regulated genes in EPQ and senescent cells. Gene sets and PI curves were determined by GRO‐seq analysis alone. (e) PI curves for 639 S_GAINED_ and 598 S_LOST_ genes in BJ fibroblast replicative senescence defined by combined RNA‐seq and GRO‐seq analysis (see methods). (f) Enriched GO terms from Metascape analysis of genes showing > 4‐fold promoter loss of H4R3me^(^^2s^^)^ in replicatively senescent versus EPQ BJ cells. (g) Heatmap of p65 ChIP‐seq enrichment in EPQ, intermediate early passage, intermediate late passage, and senescent BJ fibroblasts at promoters of S_GAINED_ genes (−/+3 kb window relative to TSS and sorted based on p65 tag count for −500 to +200 bp region in senescent cells). (h) Meta‐analysis distribution of normalized H4R3me^(2s)^ native ChIP‐seq tag density in senescent or EPQ BJ fibroblasts, centered on the TSSs of S_GAINED_‐Expanded (S_GAINED_‐EXP) genes that were segregated by the presence or absence of p65 ChIP‐seq promoter peaks in replicative senescence. (i) Table of log_2_ fold change in *CDKN1A* and *CDKN2A* expression, based on normalized RNA‐seq tag counts, in EPQ or senescent BJ fibroblasts upon shRNA knockdown of JMJD6. Change in expression is relative to shRNA negative (non‐silencing) control (shCTL) in EPQ cells. Adjusted *p*‐values are also provided. (j) Population doublings over time of BJ fibroblasts stably expressing shJMJD6 or shRNA non‐silencing (NS) control. shJMJD6−51, −53, −57, and −58 (see Figure 1f) each represent distinct shRNA constructs. (k) Box plot of log_2_ fold change in RNA‐seq tag density for genes with the greatest promoter loss of H4R3me^(2s)^ (< −2.5 log_2_ fold change in H4R3me^(2s)^ ChIP‐seq tag count in −300 to +200 bp window relative to TSS) in shJMJD6 stable knockdown versus shRNA non‐silencing control senescent BJ fibroblasts. *p* value determined by two‐tailed *z*‐test.
**Figure S2:** The functional link of H3K79me^3^ spreading across gene bodies to replicative senescence. (a) UCSC genome browser tracks of H3K79me^3^, H3K79me^2^, H2Bub and p65 distribution as well as RNA‐seq data for a representative genomic region (inclusive of the loci shown in Figure 2b) at different cell stages. Of the depicted genes, 3 are classified as S_GAINED_: TNFRSF10A, TNFRSF10C, and TNFRSF10D. (b) Box plot of log_2_ fold change in normalized H3K79me^3^ gene body tag density during replicative senescence for all BJ fibroblast‐expressed genes in listed gene‐length categories based on a ChIP‐seq biological replicate. (c) Box plot of log_2_ fold change in expression during replicative senescence for all BJ fibroblast‐expressed genes in different gene‐length categories. Values correspond to averaged log_2_ fold change in senescent vs EPQ cells based on RNA‐seq triplicates and a single GRO‐seq assay. (d) Box plot of the difference in log_2_ pausing index (PI) between senescent and EPQ BJ cells (i.e., senescent minus EPQ) for all BJ fibroblast‐expressed genes in listed gene‐length categories. (e) Representative western blot showing the effect of EPZ‐5676 dose on H3K79me^3^ levels in BJ fibroblasts. (f) Population doublings over time of BJ fibroblasts expressing doxycycline (DOX)‐inducible HA (DH)‐ or TY1 (DT)‐tagged DOT1L versus empty vector control in the continuous presence of 20 nM DOX. (g, h) Box plots of log_2_ fold change in normalized RNA‐seq tag density for either S_GAINED_ (g) or S_LOST_ (h) genes in senescent (PD 64) versus EPQ (PD 30) BJ fibroblasts upon indicated treatments. (i) Box plot of log_2_ fold change in normalized H3K79me^3^ gene body tag density during replicative senescence comparing genes with increased or decreased expression in EPZ‐5676‐treated EPQ BJ cells. (j) Box plot of log_2_ fold change in normalized H3K79me^3^ gene body tag density during replicative senescence comparing genes with increased or decreased expression in EPZ‐5676‐treated senescent (Sen) BJ cells. (k) Box plot of gene length (bp) distribution for downregulated (<−1 log_2_ fold change) and upregulated (> 1 log_2_ fold change) genes in senescent cells after prolonged EPZ‐5676 treatment. (l) Box plot of log_2_ fold change in normalized H3K79me^3^ gene body tag density during replicative senescence for genes with increased or decreased expression in EPZ‐5676‐treated senescent BJ cells. (m) Box plot of normalized GRO‐seq tags over gene bodies in untreated EPQ BJ fibroblasts for gene sets with decreased (<−1 log_2_ fold change) or increased (> 1 log_2_ fold change) expression in EPZ‐5676‐treated senescent BJ fibroblasts. (n) Box plot of normalized ChIP‐seq H3K79me^3^ promoter tags in untreated EPQ BJ fibroblasts for gene sets with decreased (<−1 log_2_ fold change) or increased (> 1 log_2_ fold change) gene expression in EPZ‐5676‐treated senescent BJ fibroblasts. For all box plots in Figure S2, *p* values were determined by two‐tailed *z*‐tests.
**Figure S3:** Association of S_GAINED_ and S_LOST_ gene sets with tissue aging. (a) Overlap of S_GAINED_ and S_LOST_ gene sets with a collection of upregulated (UAGs) and downregulated (DAGs) age‐associated genes (Jia et al. 2018). For consistency between lists, only protein‐coding genes were considered in this analysis. *p* values are for statistical enrichment using Fisher's exact test. Jaccard index indicates similarity of compared lists. Area‐proportional Venn diagrams were generated with BioVenn (Hulsen et al. 2008). (b, c) Box plots showing the distribution of gene lengths for UAGs and DAGs with overlapping S_GAINED_ and S_LOST_ genes either (b) included or (c) excluded. *p* values determined by two‐sided Mann–Whitney *U* tests. (d) Enrichment analysis of S_GAINED_ and S_LOST_ gene sets in human aging DEGs based on GTEx samples from donors aged 20–70 years using the voyAGEr webtool (Schneider et al. 2024). The selected tissues were previously reported to diverge from the typical GLTD aging trend in GTEx transcriptomic data (Stoeger et al. 2022). See Table S2 for enrichment analysis performed on all available tissues.

**Table S1:** S_GAINED_ and S_LOST_ gene sets in BJ replicative senescence.

**Table S2:** Enrichment analysis for S_GAINED_ and S_LOST_ gene sets in tissue‐stratified, age‐associated differentially expressed genes (DEGs) based on human GTEx RNA‐seq data using the voyAGEr platform (Schneider et al. 2024). Tissues highlighted in orange were inconsistent with the GLTD trend of long gene downregulation in aging according to the analysis of Stoeger et al. (2022). *p*‐values are from Fisher's exact test and each represents the ‐log_10_ (*p*‐value) for enrichment in the DEGs at a given age. *p*‐value columns are colorized from blue to yellow, indicating increasing significance within a given column, for all values > 2 (i.e., *p* < 0.01).

**Table S3:** Constructs and primers used in this study.

## Data Availability

The functional genomics sequencing data in this paper have been deposited as GEO Series GSE146585, accessible at: https://www.ncbi.nlm.nih.gov/geo/query/acc.cgi?acc=GSE146585.
