# Supplementary material for: A Histone Code Functionally Linked to Replicative Senescence
Source: Aging Cell. 2026 Jan 2;25(1):e70343. doi: 10.1111/acel.70343 (PMC12759110; doi:10.1111/acel.70343)
Supplement: Supplementary file 1 — Appendix S1: Methods and Supplementary Figures. Figure S1: Further characterization of SGAINED and SLOST genes and the functional link of H4R3me( 2s ) promoter depletion to replicative senescence. (a) Box plot of fold change in expression of SGAINED and SLOST genes during BJ fibroblast replicative senescence. Values correspond to average fold change in senescent versus EPQ cells based on RNA‐seq triplicates and a single GRO‐seq assay. p values determined by two‐tailed z‐tests. (b) Selected GO‐terms from Metascape analysis of 598 SLOST genes. Negative log10 p values for each term are plotted. (c) Box plot showing the distribution of gene lengths for SGAINED, SLOST, and unchanged gene sets in replicative senescence, as in Figure 1c, but including only protein‐coding genes. p values determined by two‐sided Mann–Whitney U tests. (d) Pausing index (PI) curves for senescent up‐ and down‐regulated genes in EPQ and senescent cells. Gene sets and PI curves were determined by GRO‐seq analysis alone. (e) PI curves for 639 SGAINED and 598 SLOST genes in BJ fibroblast replicative senescence defined by combined RNA‐seq and GRO‐seq analysis (see methods). (f) Enriched GO terms from Metascape analysis of genes showing > 4‐fold promoter loss of H4R3me( 2s ) in replicatively senescent versus EPQ BJ cells. (g) Heatmap of p65 ChIP‐seq enrichment in EPQ, intermediate early passage, intermediate late passage, and senescent BJ fibroblasts at promoters of SGAINED genes (−/+3 kb window relative to TSS and sorted based on p65 tag count for −500 to +200 bp region in senescent cells). (h) Meta‐analysis distribution of normalized H4R3me(2s) native ChIP‐seq tag density in senescent or EPQ BJ fibroblasts, centered on the TSSs of SGAINED‐Expanded (SGAINED‐EXP) genes that were segregated by the presence or absence of p65 ChIP‐seq promoter peaks in replicative senescence. (i) Table of log2 fold change in CDKN1A and CDKN2A expression, based on normalized RNA‐seq tag counts, in EPQ or senescent BJ fib [file ACEL-25-e70343-s003.pdf]

Supporting Information Appendix

A Histone Code Functionally Linked to Replicative Senescence

Thomas Suter[1,#], Meyer J. Friedman[1,#], Cagdas Tazearslan[2,#], Amir Gamliel[1], Daria Merkurjev[1], Kenneth Ohgi[1], Zhongjun Zhou[3,4], Michael G.Rosenfeld[1,*,+], Yousin Suh[2, 5, 6,*]

[1] Cellular and Molecular Medicine, Department of Medicine, University of California San Diego, La Jolla, CA, USA

[2] Department of Genetics, Albert Einstein College of Medicine, Bronx, NY, USA

[3] School of Biomedical Sciences, Li Ka Shing Faculty of Medicine, The University of Hong Kong, Hong Kong

[4] Shenzhen Institute of Innovation and Research, The University of Hong Kong, Nanshan, Shenzhen, China

[5] Department of Obstetrics and Gynecology, Columbia University Irving Medical Center, New York, NY, USA

[6] Department of Genetics and Development, Columbia University Irving Medical Center, New York, NY, USA

[#] equal contributions

+ Lead Contact

[*]To whom correspondence should be addressed:

Yousin Suh: ys3214@cumc.columbia.edu;

Michael G. Rosenfeld: mrosenfeld@health.ucsd.edu

Appendix includes:

Methods
Figure S1-S3

Methods

Tissue culture

BJ fibroblasts (ATCC® CRL-2522) were cultured at 37 °C in 20% O_2 and 5% CO_2 in high-glucose DMEM (4.5 g/L , Gibco 10566-016) supplemented with 10% FBS. Except where indicated otherwise, fibroblasts were initially plated at population doubling (PD) 26. Cells were grown to confluence and then split 1:8. PD was incremented by 3 upon each 1:8 passage.

BJ fibroblasts were deemed "early passage" when cultured below PD 35. Intermediate fibroblasts were harvested between PD 35-50. Fibroblasts were considered senescent when they failed to divide within one week of passaging, which typically occurred after at least PD 64 and was initially confirmed by beta-galactosidase staining.

To limit cell cycle effects, non-senescent, dividing fibroblasts were grown to confluence prior to harvesting for any experiments, resulting in contact inhibited quiescence and similar growth arrest among all populations of cells. Early passage cells collected in this manner are referred to as early passage quiescent (EPQ).

Short-term shRNA experiments were performed by transducing senescent (>PD 60) BJ fibroblasts with pLKO.1 or GIPZ lentivirus. Cells were maintained under selection with 0.6 µg/ml puromycin starting 4 days after transduction and continuing until harvesting, 14 days after transduction.

For long-term shRNA experiments, early passage (<PD 30) BJ fibroblasts were transduced with pLKO.1 or GIPZ lentivirus, maintained under selection with 0.6 µg/ml puromycin while expanding for 2 weeks, and then cryopreserved upon harvesting. After thawing, frozen stable lines were grown continuously until reaching senescence, and their population doublings were recorded over time. Both early passage and senescent stable knockdown lines were harvested for further experiments.

Tet-inducible overexpression experiments involved transducing early passage (<PD 30) BJ fibroblasts with a cocktail of pRetroX and TetOn retroviruses. Beginning 3 days after transduction, cells were selected with 0.6 µg/ml puromycin (Gibco A1113803) and 0.3mg/ml G418 (GeminiBio 400-113). Cells were expanded for 2 weeks under selection prior to cryopreservation. After thawing, frozen stable lines were grown in the continuous presence of 20nM doxycycline (for sustained expression of the transgene), and population doublings were recorded.

For long term EPZ-5676 DOT1L inhibitor treatment, early passage BJ fibroblasts were maintained in DMSO, 10nM rapamycin, 500nM EPZ-5676, or a combination of 10nM rapamycin and 500nM EPZ-5676. Cell population doublings were recorded, and cells were harvested for RNA-seq as well as ChIP-seq at EPQ, intermediate quiescent, and senescent stages.

Virus production and transduction conditions

Lentivirus was produced by transfection of packaging plasmids into 293T cells (ATCC® CRL-3216) at ~80% confluency, while plasmids used for generation of retrovirus for dox-inducible overexpression of histone modifying factors were transfected into the Gryphon amphitropic retroviral packaging cell line (Allele Biotechnology ABP-RVC-10001). Lipofectamine 2000 (Thermo Fisher 12566014) was used for all transfections as per manufacturer's protocol.

Retrovirus for tetracycline-inducible expression was generated by transfecting 11 µg of either the TetOn or pRetroX plasmid into a 10cm dish, using 35 µl of Lipofectamine 2000. Lentivirus for shRNA knockdown experiments was generated by transfecting a 0.625 : 1.875 : 2.5 µg ratio of MD2.G envelope plasmid: psPAX2 packaging plasmid: pGIPZ/pLKO.1 shRNA transfer plasmid with 17 µl Lipofectamine 2000 in a single well of a 6-well plate. Media was changed 6 hours post transfection, and the virus-containing media was collected at 48 and 72 hours post transfection. Virus-containing media was centrifuged at 600xg for 5 min, and the viral supernatant was collected and stored at 4°C prior to transduction.

Retroviral transduction was performed on actively growing BJ fibroblasts at ~50% confluency. Lentiviral transduction was performed on both early passage and senescent BJ fibroblasts at ~50% confluency. For generation of tet-responsive overexpression lines, a 1:1:1 ratio of pRetroX, TetOn viral supernatants and culture media was used. All transduction mixtures were added to cells overnight with polybrene (8 µg/ml final concentration). Dox-responsive overexpression lines were maintained in 0.6 µg/ml puromycin (Gibco A1113803) and 0.3mg/ml G418 (GeminiBio 400-113) starting 4 days after transduction.

GIPZ stable knockdown lines were generated using a 667 µl : 1333 µl ratio of GIPZ viral supernatant to fresh culture media. pLKO.1 stable knockdown lines were generated using a 48 µl : 1952 µl ratio of pLKO.1 viral supernatant to fresh culture media. All transduction mixtures were added to cells overnight with 8 µg/ml polybrene. Knockdown cells were maintained in 0.6 µg/ml puromycin starting 4 days after transduction. All shRNA knockdowns were validated via qPCR to elicit >75% reduction in mRNA targets.

Plasmid constructs

Stable knockdown of gene transcripts was performed with pLKO.1 and pGIPZ vectors expressing short hairpin RNAs (shRNAs). The shRNA constructs targeting JMJD6 were purchased from the Albert Einstein College of Medicine shRNA Core Facility (see **Table S3** for acquired plasmids).

Constructs for stable inducible expression of JMJD6, PRMT1, PRMT5, and DOT1L were generated by cloning ORFs into the pRetroX-Tight-Puro Dox Inducible expression vector (Clontech). ORF constructs for JMJD6, PRMT1, and PRMT5 were purchased from Albert Einstein College of Medicine shRNA Core Facility (**Table S3**). The DOT1L ORF, corresponding to NM_032482.3, was cloned from a construct previously generated in our lab. For each gene, two separate overexpression constructs, one with an N terminal 2X-HA/2X-FLAG tag and one with a C-terminal 2X-TY1 tag, were made.

The 2X-HA/2X-FLAG construct was generated by synthesizing 2 oligos containing a Kozak sequence followed by the epitope tags and flanked by BamHI-compatible overhangs. The 3' end was designed to reconstitute a BamHI site upon ligation with pRetroX, whereas the 5' end would destroy the BamHI recognition site. Annealed oligos were ligated into the BamHI-digested pRetroX empty vector. The ORFs were then amplified with PCR primers containing NotI and MluI restriction sites, digested with these restriction enzymes, and ligated into the NotI-/MluI-digested 2XHA/2XFLAG-tagged pRetroX vector. The 2X-TY1 constructs were generated by amplifying each ORF with 5'-primers containing a NotI restriction site and a Kozak sequence and 3'-primers containing a MluI restriction site and a 2X-TY1 tag. The ORFs were then amplified with PCR primers containing NotI and MluI restriction sites, digested with these restriction enzymes, and ligated into the NotI-/MluI-digested empty pRetroX vector (see **Table S3** for primer sequences).

RNA-seq

Total cellular RNA was harvested from cells using the RNeasy Mini Kit (QIAGEN-74104), including the DNase treatment step. Samples were prepared for sequencing using the TruSeq Stranded Total RNA LT Sample Prep Kit (RS-122-2201/RS-122-2202) for ribosomal RNA-depleted libraries. Samples were run on the Illumina HiSeq 4000 as single-read reactions of 50 or 75 cycles.

ChIP with fixation

Fixed chromatin immunoprecipitation assays were performed as previously described (Li et al., 2013) with some modifications. Samples for histone modification ChIPs were fixed in 1% formaldehyde for 10 minutes at room temperature. Samples for transcription factor ChIPs were double cross-linked at room temperature in 2mM DSG for 45 minutes followed by 10-minute fixation with 1% formaldehyde. After

neutralization with 0.125M glycine, nuclei were isolated and resuspended in nuclear lysis buffer (50mM Tris-HCl pH7.4, 1% SDS, 10mM EDTA, 1x cOmplete Protease Inhibitor Cocktail (Sigma 11836145001)), and then sonicated using the Bioruptor (Diagenode). The concentration of chromatin in each sample was determined using the Qubit Fluorometer (Thermo Fisher), and samples were then diluted 1:10 in dilution buffer (20mM Tris/HCl pH7.4, 100mM NaCl, 0.5% Triton-X-100, 2mM EDTA, 1x cOmplete Protease Inhibitor Cocktail). For each histone ChIP assay, 20-50 µg of chromatin from each sample was diluted in a 1:10 lysis:dilution buffer for a final volume of 0.5 mL. Samples were incubated with antibodies on a rotator at 4°C overnight.

Antibody:chromatin complexes were immunoprecipitated as described using 20 µl Protein G Dynabeads (Thermo Fisher). ChIPs of histone modifications were washed with the following buffers/conditions in the indicated order: 2xWB1 (20mM Tris/HCl pH7.4, 150mM NaCl, 1% Triton-X-100, 0.1% SDS, 2mM EDTA), 1xWB2 (20mM Tris/HCl pH7.4, 500mM NaCl, 1% Triton-X-100, 2mM EDTA), 1xWB3 (10mM Tris/HCl pH7.4, 250mM LiCl, 1% NP-40, 1% Deoxycholate, 1mM EDTA), 2xTE (10mM Tris/HCl pH 8.0, 1mM EDTA). After washing steps, samples were eluted in EB (1% SDS, 0.1M $NaHCO_3$) at 65 °C, followed by decrosslinking overnight at 65 °C, and then purified using the QIAquick PCR Purification Kit (QIAGEN). Finally, samples were further sonicated for 10 minutes to ensure the majority of purified chromatin was 100-300bp.

Purified chromatin for histone ChIPs was prepared for deep sequencing with the KAPA LTP Library Preparation Kit (KK8232), following manufacturer's instructions. Samples were run on the Illumina HiSeq 2500 as single-read reactions of 50 or 75 cycles.

Native ChIP

Native ChIP of H4R3me2s was performed as described in a previous report (Wagschal et al., 2007), which is summarized below, with alterations to the original protocol noted. 5-10 million BJ fibroblasts, either senescent or quiescent via contact inhibition, were harvested via trypsinization, with care taken to ensure that cells were in a single-cell suspension. Cells were transferred to a 15ml conical tube and pelleted at 3000xg for 5 minutes at 4°C. All subsequent steps, unless otherwise noted, were performed at 4°C with cold buffers. Cells were resuspended in 2ml nuclei preparation buffer I (0.3M sucrose, 60mM KCl, 15mM NaCl, 5mM MgCl2, 0.1mM EGTA, 15mM Tris-HCl pH 7.5, 0.5mM DTT, 1x dissolved cOmplete Protease Inhibitor Cocktail), mixed gently with 2ml of nuclei preparation buffer II (0.3M sucrose, 60mM KCl, 15mM NaCl, 5mM MgCl2, 0.1mM EGTA, 15mM Tris-HCl pH 7.5, 0.5mM DTT, 1x dissolved cOmplete Protease Inhibitor Cocktail, 0.4% NP-40), and then incubated on ice for a maximum of 5 minutes. 8 ml of nuclei

preparation buffer III was added to 2 new 15ml polypropylene tubes. 2ml of the 4ml cell suspension was layered on top of each 8ml sucrose cushion and tubes were centrifuged in a pre-chilled swing-out rotor at 10,000xg for 20 minutes at 4°C. The supernatant was removed completely, and the nuclear pellet was resuspended in 1ml of MNase digestion buffer (0.32M sucrose, 4mM MgCl2, 1mM CaCl2, 50mM Tris-HCl pH 7.5, 0.1 mM PMSF), before splitting into two 500 µl aliquots in separate 1.5ml Eppendorf tubes. 1 µl of MNase enzyme (NEB-M0247S) was added to each tube, and the tubes were incubated for 2 and 5 minutes at 37°C. The reactions were stopped by addition 5 µl of 0.5M EDTA and 5 µl of 0.5M EGTA to each, and tubes were then chilled on ice. Each tube was centrifuged for 10,000 rpm for 10 minutes at 4°C and the supernatants, containing the first fraction of soluble chromatin, were collected. Each remaining nuclear pellet was resuspended in 500 µl dialysis-lysis buffer (1mM Tris-HCl pH 7.5, 0.2 mM PMSF, 0.2mM EDTA), incubated for 1 hour at 4°C, and then re-centrifuged at 10,000 rpm for 10 minutes at 4°C. The supernatants, containing the second fraction of soluble chromatin, were collected. Concentrations of each fraction were measured via Qubit, and the fragment size distribution of each fraction was checked by agarose gel electrophoresis to confirm similar digestion between each biological sample. Fractions were then diluted in their respective buffers to ensure a similar concentration of chromatin. Equal volumes of each fraction for each biological sample were pooled and diluted so that each ChIP reaction contained ~10 µg of chromatin in 500 µl total volume, which was equal parts MNase digestion buffer and dialysis-lysis buffer with equivalent amounts of each chromatin fraction. 1ml of ChIP incubation buffer (50mM Tris-HCl, pH 7.5, 5mM EDTA, 50mM NaCl, 1x dissolved cOmplete Protease Inhibitor Cocktail) was added to each 500 µl ChIP reaction. Following addition of 5 µg of antibody to each reaction, tubes were rotated overnight at 4°C. In parallel, 20 µl of Protein G Dynabeads (Thermo Fisher) per ChIP reaction were washed and blocked overnight in 1% BSA/glycogen in ChIP incubation buffer, prior to resuspension in their original volume with ChIP incubation buffer.

After overnight incubation, 20 µl of blocked beads was added to each ChIP reaction and incubated with rotation for 4 hours at 4°C. Beads were then washed, using the same magnetic bead washing protocol as with the fixed ChIP reactions: 2 times with Washing Buffer A (50mM Tris-HCl, pH 7.5, 10mM EDTA, 75mM NaCl), 2 times with Washing Buffer B (50mM Tris-HCl, pH 7.5, 10mM EDTA, 125mM NaCl), and 2 times with Washing Buffer C (50mM Tris-HCl, pH 7.5, 10mM EDTA, 175mM NaCl). Following washing steps, beads were resuspended in 48 µl Proteinase K digestion buffer with 2 mg/ml Proteinase K and incubated for 30 minutes at 50°C. Supernatants were purified with the QIAquick PCR Purification Kit (QIAGEN) and

eluted in EB. Samples were sonicated for 10 minutes in the Bioruptor to ensure that immunoprecipitated chromatin from polynucleosomes was 100-300bp in size for sequencing.

Libraries were prepared for sequencing as with formaldehyde-fixed ChIPs, using the KAPA LTP Library Preparation Kit (KK8232), according to manufacturer's instructions. Samples were run on the Illumina HiSeq 2500 as single-read reactions of 50 cycles.

Antibodies

The following antibodies were used in this publication: HA, Abcam ab9110; TY1, Diagenode C15200054; p65, Santa Cruz sc-372 (C-20); H3K4me2, Abcam ab7766; H3K27ac, Abcam ab4729; H4R3me2s, Millipore 17-10250 (ChIP-seq) and Abcam ab5823 (Western blot); H3K79me3, Abcam ab2621; H3K79me2, Abcam ab3594; H2Bub, Millipore CS200595; H4R3me2as, Active Motif 39705; H3K4me1, Abcam ab8895; H3K4me3, Millipore 07-473; H3K9me3, Active Motif 39161; H3K27me3, Millipore 07-449; H3K36me2, Active Motif 39255; H3, Abcam ab1791; H4, Abcam ab 7311; H3K36me3, Abcam ab9050; H3K79me1, Abcam ab2886; H3.3, Abcam ab4263; H4ac, Millipore 06-598; H4K8ac, Millipore 07-328; H4K16ac, Abcam ab61240; H4K20me3, Abcam ab9053; H1K25me2, Abcam ab20652.

Histone western blot

Histones were extracted for western blot analysis using the Abcam protocol. Cells were harvested via trypsinization and washed in ice-cold 1X PBS supplemented with 5mM sodium butyrate. Cells were resuspended in Triton Extraction buffer (PBS containing 0.5% Triton X-100, 2mM PMSF, 0.02% NaN_3) at a cell density of 10^7 cells per ml and lysed on ice for 10 minutes with gentle stirring. Each sample was then centrifuged at 2000rpm for 10 minutes at 4°C and supernatants were discarded. The pellets were washed and re-centrifuged once in half the previous volume of Triton Extraction Buffer. The pellets were then resuspended in 0.2N HCl at $4x10^7$ cells per ml and incubated overnight at 4°C. Samples were centrifuged at 2000rpm for 10 minutes at 4°C, and the supernatant was collected for western blotting using a XCell SureLock Mini-Cell and XCell II Blot Module (ThermoFisher EI0002).

Samples were boiled in NuPAGE LDS Sample Buffer (ThermoFisher NP0007) and resolved on 1.5 mm, 15-well NuPAGE 4-12% Bis-Tris Protein Gels (ThermoFisher NP0336BOX) using 1x NuPAGE MES SDS Running Buffer (20x ThermoFisher NP0002). Samples were transferred onto nitrocellulose membranes for >2 hours using 1X NuPage Transfer Buffer (ThermoFisher NP00061). The amount of histone material in each well was initially assessed by Ponceau S staining and blotting of total histone H3 or H4 levels. Loading amounts were adjusted accordingly, and samples were rerun. Loading of normalized samples was

confirmed with Ponceau S staining. Membranes were blocked with 5% milk and incubated with primary antibodies at 4°C overnight. After washing with 1X PBS containing 0.01% Tween-20 (PBS-T), membranes were incubated with HRP-conjugated secondary antibody at room temperature, washed again with PBS-T, and ultimately imaged via chemiluminescence upon exposure to film.

Global Run-On sequencing (GRO-seq)

GRO-seq was performed as described previously (Li et al., 2013). In brief, approximately 10 million BJ fibroblasts (either senescent or quiescent) were used per experimental condition. Cells were incubated for 5 minutes on ice in swelling buffer (10mM Tris-Cl pH7.5, 2mM MgCl2, 3mM CaCl2), lysed in lysis buffer (swelling buffer with 0.5% IGEPAL and 10% glycerol), and finally re-suspended in 100ul freezing buffer (50mM Tris-Cl pH8.3, 40% glycerol, 5mM MgCl2, 0.1mM EDTA). The run-on reaction was then performed by mixing the re-suspended nuclei with an equal volume of run-on reaction buffer (10mM Tris-Cl pH 8.0, 5mM MgCl2, 1mM DTT, 300mM KCl, 20 units of Superase-In, 1% sarkosyl, 500µM ATP, 500µM GTP, 500µM Br-UTP, and 2µM CTP) and incubating tubes for 5 min in a 30°C water bath. Nuclear run-on RNA (NRO-RNA) was extracted with TRIzol LS reagent (Invitrogen), as per manufacturer's instructions. NRO-RNA was hydrolyzed to ~300bp fragments via 35-minute incubation on ice in 0.2M NaOH, which was followed by treatment with DNase I and Antarctic phosphatase. Br-UTP labeled NRO-RNA was purified with anti-BrdU agarose beads (Santa Cruz Biotech) in binding buffer (0.5×SSPE, 1mM EDTA, 0.05% tween) by rotating samples at 4°C for 3h. T4 PNK (NEB) was then used to end-repair isolated NRO-RNA.

RNA fragments were incubated with poly-A polymerase (NEB) for 30 min at 37°C, after which reverse transcription was performed using superscript III (Invitrogen) and the oNTI223 primer (**Table S3**). cDNA products were run on a 10% polyacrylamide TBE-urea gel, and products between 100-500bp were excised and recovered by gel extraction. First strand cDNA was circularized via CircLigase (Epicentre) and then re-linearized by treatment with Ape1 (NEB). The re-linearized cDNA was then size selected on a TBE gel, and fragments between ~120-320 bp were recovered via gel extraction. Finally, this cDNA was PCR amplified using Phusion High-Fidelity enzyme (NEB) with oNTI200 ID1-6 (each sample was amplified with a different ID# primer to impart a distinct barcode) and oNTI201 (**Table S3**). Samples were sequenced on the HiSeq 2500 system with single read runs of 50 cycles.

Processing of deep sequencing data

All replicates passing FASTQC were included in analyses. Deep sequencing reads were aligned to the hg18 assembly.

ChIP-seq data were aligned using STAR-aligner with parameters adjusted to ignore spliced alignments, and aligned reads were processed for downstream analysis using the HOMER (Heinz et al., 2010) "makeTagDirectory" program, filtering for tags that were uniquely mapped and only selecting at most one sequencing read per position per length of read. Histone peaks were identified using the HOMER "findPeaks" program with the "-histone" style parameter in order to identify broader peaks and stitch together peaks of less than 1000bp.

Quantitation of ChIP-seq signal was done using the "annotatePeaks.pl" program in HOMER with the default normalization to 10 million tags. Meta-analyses and heatmap analyses of ChIP-seq data also employed the "annotatePeaks.pl" program in HOMER, using the subset of genomic coordinates relevant to the given conditions in each figure. Box plots of H4R3me$^{(2s)}$ promoter-enrichment and H3K79me^{3} gene-body density were not median normalized, so as to preserve the dramatic differences in the total levels of these marks during senescence, as indicated by western blot analysis.

RNA-seq and GRO-seq data were aligned using STAR-aligner, with parameters adjusted to account for spliced alignment. Aligned reads were processed for downstream analysis using the HOMER "makeTagDirectory" program, filtering for uniquely mapped tags and selecting at most one sequencing read per position per length of read. A preliminary set of transcript coordinates was generated by selecting the coordinates of the longest isoform for each gene. The RNA-seq and GRO-seq datasets in EPQ and senescent conditions were quantified over these preliminary transcript coordinates using the HOMER "annotatePeaks.pl" program. Transcripts with an average of less than 7 tags in either RNA-seq or GRO-seq runs of a combination of EPQ and senescent preliminary experiments were discarded, leaving 14493 transcripts above our expression threshold that were used for downstream analysis.

Processed RNA-seq and GRO-seq datasets were quantified over this transcript list representing 14493 genes using the HOMER "annotatePeaks.pl" program. The normalized $\log_2$ fold change in tag density was determined between each condition being compared for all 14493 genes, which was then median normalized for each comparison so that the median $\log_2$ fold change in tag density for all 14493 transcripts between two experiments would be zero. Box plots represent the distribution of these median-normalized fold changes over various listed subsets of the 14493 gene transcripts. "Averaged" fold changes in transcription were obtain by averaging all replicates of RNA-seq and GRO-seq performed in the listed condition.

Pausing index was calculated as the ratio of the tag density in the first 1000bp of each transcript relative to the tag density in the remaining portion of the respective gene. Gene lengths were based on Refseq reported gene coordinates.

Box plot information

Box plots indicate the population median value (indented center line), 25th and 75th percentiles (bottom and top edges of boxes, respectively), and, with the exception of those panels noted below, 5th and 95th percentiles (ends of lower and upper whiskers, respectively). For box plots in Fig. 1c as well as Fig. S1c, S3b, and S3c, lower and upper whiskers represent minimum and maximum values, respectively.

Determination of gene subsets

The genes considered up- or down-regulated in senescence (S_{GAINED} or S_{LOST}) were at least 1 standard deviation beyond a 1.5-fold change in paired RNA-seq and GRO-seq datasets. 3 RNA-seq replicates paired with a single GRO-seq experiment from EPQ vs senescent BJ fibroblasts were used to determine S_{GAINED} and S_{LOST} genes. The S_{GAINED}-EXP gene set is an expanded set of S_{GAINED} genes that was used for meta-analysis plots of H4R3me$^{(2s)}$, as it provided a smoother representation of the ChIP data to account for the weaker enrichment in this particular assay. The S_{GAINED}-EXP gene set, comprising 2621 genes showing a fold change greater than 2 standard deviations above zero, were based on the same 3 RNA-seq and 1 GRO-seq paired datasets of EPQ vs senescent BJ fibroblasts used for S_{GAINED} and S_{LOST} gene designation.

Significance testing of gene populations

To determine whether different sets of distributions of fold changes, including those generated for RNA-seq, GRO-seq, and ChIP-seq and typically shown in box plot figures, are significantly different from either each other or from zero (i.e., that the distributions either do or do not show a significant directional change), two-tailed z-tests were performed using the median-normalized $\log_2$ fold change in tag densities (either RNA-seq, GRO-seq, or ChIP-seq quantified and median normalized over transcript coordinates as described above). For gene length distributions, unless otherwise noted, two-sided Mann–Whitney U tests were used to determine significant differences. The output p-values are reported in each graph next to their respective comparisons. Population sizes are defined by the gene set indicated in each panel and described in more detail above.

Normalization of H3K79me^3 tag density across gene bodies

To calculate the normalized tag density of H3K79me^3 across gene bodies, HOMER was used to determine the total number of tags mapped to each gene (normalized by the tags per experiment), which was divided by the length of each gene to yield the "raw" tags/bp. For each H3K79me^3 experiment, the "background" tags/bp was calculated by determining the H3K79me^3 tags/bp of a set of universally "non-expressed" genes (in the conditions assayed for BJ fibroblasts). This "non-expressed" gene set of 1254 genes, consists of genes >15kb, minus the 14493 "expressed" gene set, minus genes showing any H3K79me^3 peaks, and minus genes above additional stringent RNA-seq and GRO-seq expression density thresholds. The determination of background tags/bp at these genes also excluded 10kb upstream of the TSS to prevent the possibility of promoter H3K79me^3 raising the background tags/bp. The "normalized" H3K79me^3 for each gene/experiment pairing was determined by subtracting the "background" tags/bp from the "raw" tags/bp for each gene in each experiment.

Analysis of H4R3me$^{(2s)}$ promoter tags

As promoter H4R3me$^{(2s)}$ was found by meta-analysis to be enriched in EPQ cells in a narrow peak positioned at approximately -300 to +200 bp, relative to the gene TSS, only this window was used in the determination of fold change in promoter-associated native H4R3me$^{(2s)}$ ChIP-seq enrichment for EPQ, senescent, and JMJD6-knockdown experiments. To determine whether H4R3me$^{(2s)}$ promoter depletion in replicatively senescent cells was specific to those with p65 occupancy, HOMER was first used to identify p65 ChIP-seq peaks that overlap with S_{GAINED}-EXP gene promoters (-/+ 0.5kb relative to the TSS) in the senescent condition. H4R3me$^{(2s)}$ tag density analysis in EPQ and senescent cells was then performed separately for p65-binding and p65-nonbinding S_{GAINED}-EXP gene promoters using HOMER.

References

Heinz, S. *et al.* Simple combinations of lineage-determining transcription factors prime cis-regulatory elements required for macrophage and B cell identities. *Mol. Cell* **38**, 576–589 (2010).

Li, W. *et al.* Functional roles of enhancer RNAs for oestrogen-dependent transcriptional activation. *Nature* **498**, 516–520 (2013).

Wagschal, A., Delaval, K., Pannetier, M., Arnaud, P. & Feil, R. Chromatin Immunoprecipitation (ChIP) on Unfixed Chromatin from Cells and Tissues to Analyze Histone Modifications. *Cold Spring Harb Protoc* **2007**, pdb.prot4767 (2007).

Figure S1

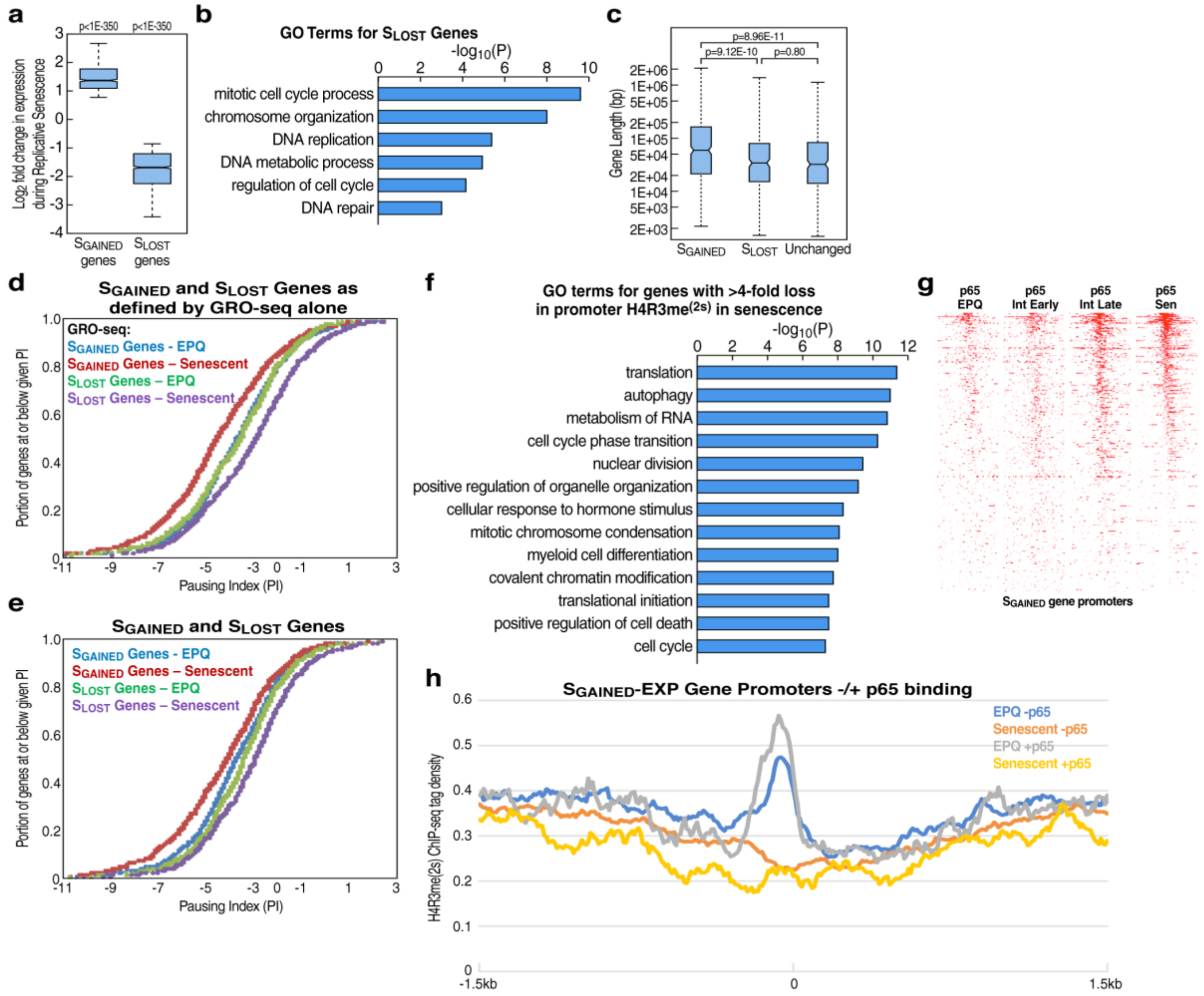

i

	Log2-fold change in CDKN1A	Adjusted p-value	Log2-fold change in CDKN2A	Adjusted p-value
EPQ, shJMJD6	0.78060309	0.24715949	-0.6927122	0.34422194
SEN, shCTL	1.31892897	0.02229822	-1.1349785	0.07347137
SEN, shJMJD6	0.3585641	0.74145349	-0.6005656	0.49024367

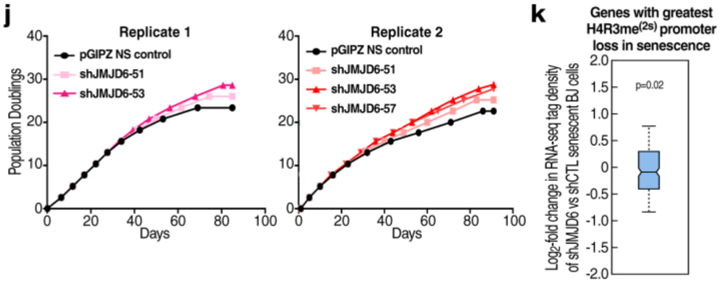

Suter et al., 2025b, S1

Figure S1. Further characterization of SGAINED and SLOST genes and the functional link of H4R3me(2s) promoter depletion to replicative senescence. (a) Box plot of fold change in expression of SGAINED and SLOST genes during BJ fibroblast replicative senescence. Values correspond to average fold change in senescent versus EPQ cells based on RNA-seq triplicates and a single GRO-seq assay. *p* values determined by two-tailed *z*-tests. (b) Selected GO-terms from Metascape analysis of 598 SLOST genes. Negative $\log_{10} p$ values for each term are plotted. (c) Box plot showing the distribution of gene lengths for SGAINED, SLOST, and unchanged gene sets in replicative senescence, as in Figure 1c, but including only protein-coding genes. *p* values determined by two-sided Mann–Whitney *U* tests. (d) Pausing index (PI) curves for senescent up- and down-regulated genes in EPQ and senescent cells. Gene sets and PI curves were determined by GRO-seq analysis alone. (e) PI curves for 639 SGAINED and 598 SLOST genes in BJ fibroblast replicative senescence defined by combined RNA-seq and GRO-seq analysis (see methods). (f) Enriched GO terms from Metascape analysis of genes showing > 4-fold promoter loss of H4R3me(2s) in replicatively senescent versus EPQ BJ cells. (g) Heatmap of p65 ChIP-seq enrichment in EPQ, intermediate early passage, intermediate late passage, and senescent BJ fibroblasts at promoters of SGAINED genes (−/+3 kb window relative to TSS and sorted based on p65 tag count for −500 to +200 bp region in senescent cells). (h) Meta-analysis distribution of normalized H4R3me(2s) native ChIP-seq tag density in senescent or EPQ BJ fibroblasts, centered on the TSSs of SGAINED-Expanded (SGAINED-EXP) genes that were segregated by the presence or absence of p65 ChIP-seq promoter peaks in replicative senescence. (i) Table of $\log_2$ fold change in *CDKN1A* and *CDKN2A* expression, based on normalized RNA-seq tag counts, in EPQ or senescent BJ fibroblasts upon shRNA knockdown of JMJD6. Change in expression is relative to shRNA negative (non-silencing) control (shCTL) in EPQ cells. Adjusted *p*-values are also provided. (j) Population doublings over time of BJ fibroblasts stably expressing shJMJD6 or shRNA non-silencing (NS) control. shJMJD6−51, −53, −57, and −58 (see Figure 1f) each represent distinct shRNA constructs. (k) Box plot of $\log_2$ fold change in RNA-seq tag density for genes with the greatest promoter loss of H4R3me(2s) ($< -2.5 \log_2$ fold change in H4R3me(2s) ChIP-seq tag count in −300 to +200 bp window relative to TSS) in shJMJD6 stable knockdown versus shRNA non-silencing control senescent BJ fibroblasts. *p* value determined by two-tailed *z*-test.

Figure S2

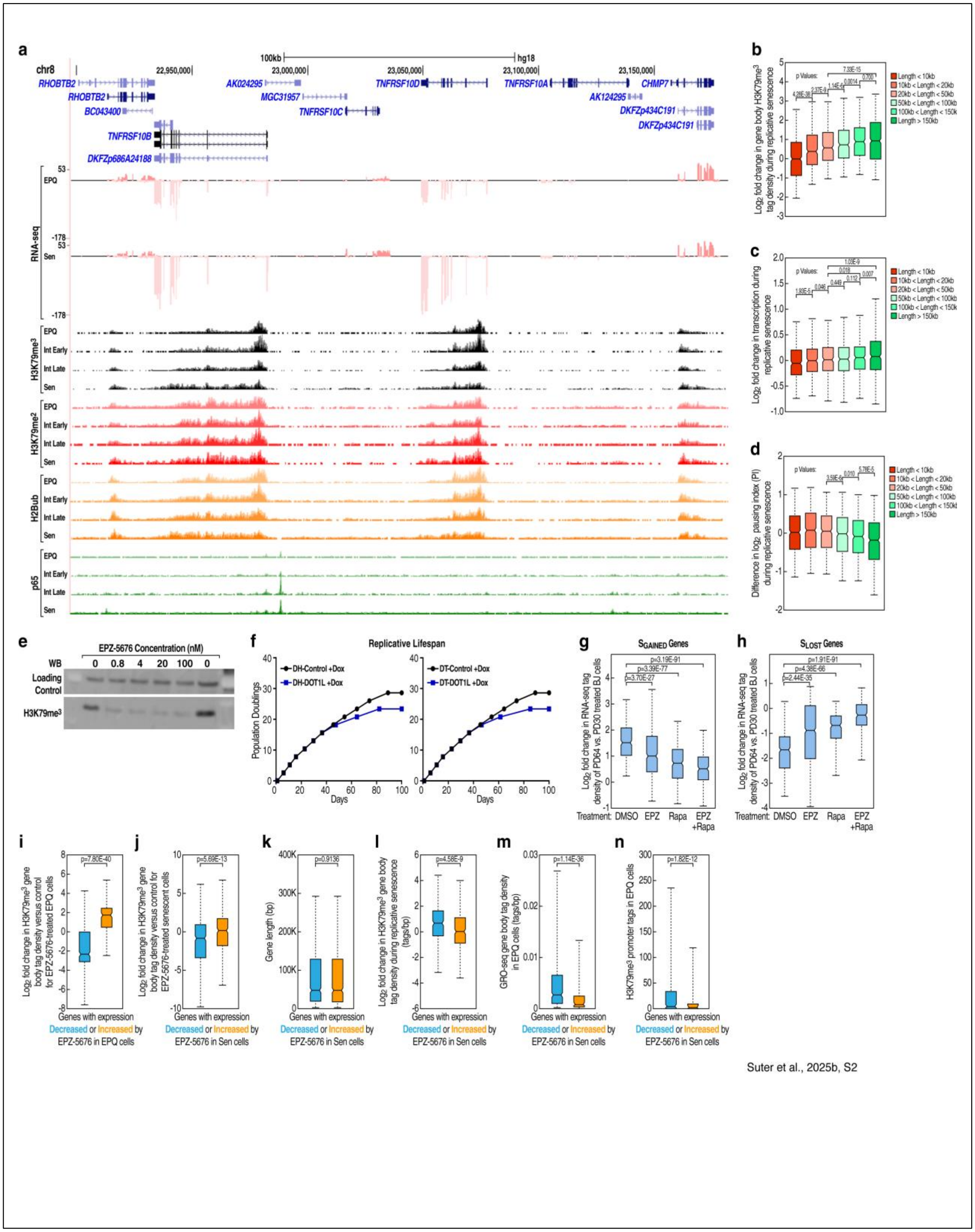

a
b
c
d
e
f
g
h
i
j
k
l
m
n
chr8
RNA-seq
H3K79me3
H3K79me2
H2Bub
p65
EPQ
Int Early
Int Late
Sen
EPZ-5676 Concentration (nM)
Loading Control
Replicative Lifespan
Population Doublings
Days
DH-Control +Dox
DH-DOT1L +Dox
DT-Control +Dox
DT-DOT1L +Dox
S GAINED Genes
S LOST Genes
Treatment: DMSO EPZ Rapa EPZ +Rapa
Genes with expression Decreased or Increased by EPZ-5676 in EPQ cells
Genes with expression Decreased or Increased by EPZ-5676 in Sen cells

Figure S2. The functional link of $H3K79me^3$ spreading across gene bodies to replicative senescence. (a) UCSC genome browser tracks of $H3K79me^3$, $H3K79me^2$, H2Bub and p65 distribution as well as RNA-seq data for a representative genomic region (inclusive of the loci shown in Figure 2b) at different cell stages. Of the depicted genes, 3 are classified as S_{GAINED}: TNFRSF10A, TNFRSF10C, and TNFRSF10D. (b) Box plot of $\log_2$ fold change in normalized $H3K79me^3$ gene body tag density during replicative senescence for all BJ fibroblast-expressed genes in listed gene-length categories based on a ChIP-seq biological replicate. (c) Box plot of $\log_2$ fold change in expression during replicative senescence for all BJ fibroblast-expressed genes in different gene-length categories. Values correspond to averaged $\log_2$ fold change in senescent vs EPQ cells based on RNA-seq triplicates and a single GRO-seq assay. (d) Box plot of the difference in $\log_2$ pausing index (PI) between senescent and EPQ BJ cells (i.e., senescent minus EPQ) for all BJ fibroblast-expressed genes in listed gene-length categories. (e) Representative western blot showing the effect of EPZ-5676 dose on $H3K79me^3$ levels in BJ fibroblasts. (f) Population doublings over time of BJ fibroblasts expressing doxycycline (DOX)-inducible HA (DH)- or TY1 (DT)-tagged DOT1L versus empty vector control in the continuous presence of 20 nM DOX. (g, h) Box plots of $\log_2$ fold change in normalized RNA-seq tag density for either S_{GAINED} (g) or S_{LOST} (h) genes in senescent (PD 64) versus EPQ (PD 30) BJ fibroblasts upon indicated treatments. (i) Box plot of $\log_2$ fold change in normalized $H3K79me^3$ gene body tag density during replicative senescence comparing genes with increased or decreased expression in EPZ-5676-treated EPQ BJ cells. (j) Box plot of $\log_2$ fold change in normalized $H3K79me^3$ gene body tag density during replicative senescence comparing genes with increased or decreased expression in EPZ-5676-treated senescent (Sen) BJ cells. (k) Box plot of gene length (bp) distribution for downregulated (<-1 $\log_2$ fold change) and upregulated (> 1 $\log_2$ fold change) genes in senescent cells after prolonged EPZ-5676 treatment. (l) Box plot of $\log_2$ fold change in normalized $H3K79me^3$ gene body tag density during replicative senescence for genes with increased or decreased expression in EPZ-5676-treated senescent BJ cells. (m) Box plot of normalized GRO-seq tags over gene bodies in untreated EPQ BJ fibroblasts for gene sets with decreased (<-1 $\log_2$ fold change) or increased (> 1 $\log_2$ fold change) expression in EPZ-5676-treated senescent BJ fibroblasts. (n) Box plot of normalized ChIP-seq $H3K79me^3$ promoter tags in untreated EPQ BJ fibroblasts for gene sets with decreased (<-1 $\log_2$ fold change) or increased (> 1 $\log_2$ fold change) gene expression in EPZ-5676-treated senescent BJ fibroblasts. For all box plots in Figure S2, p values were determined by two-tailed z-tests.

Figure S3

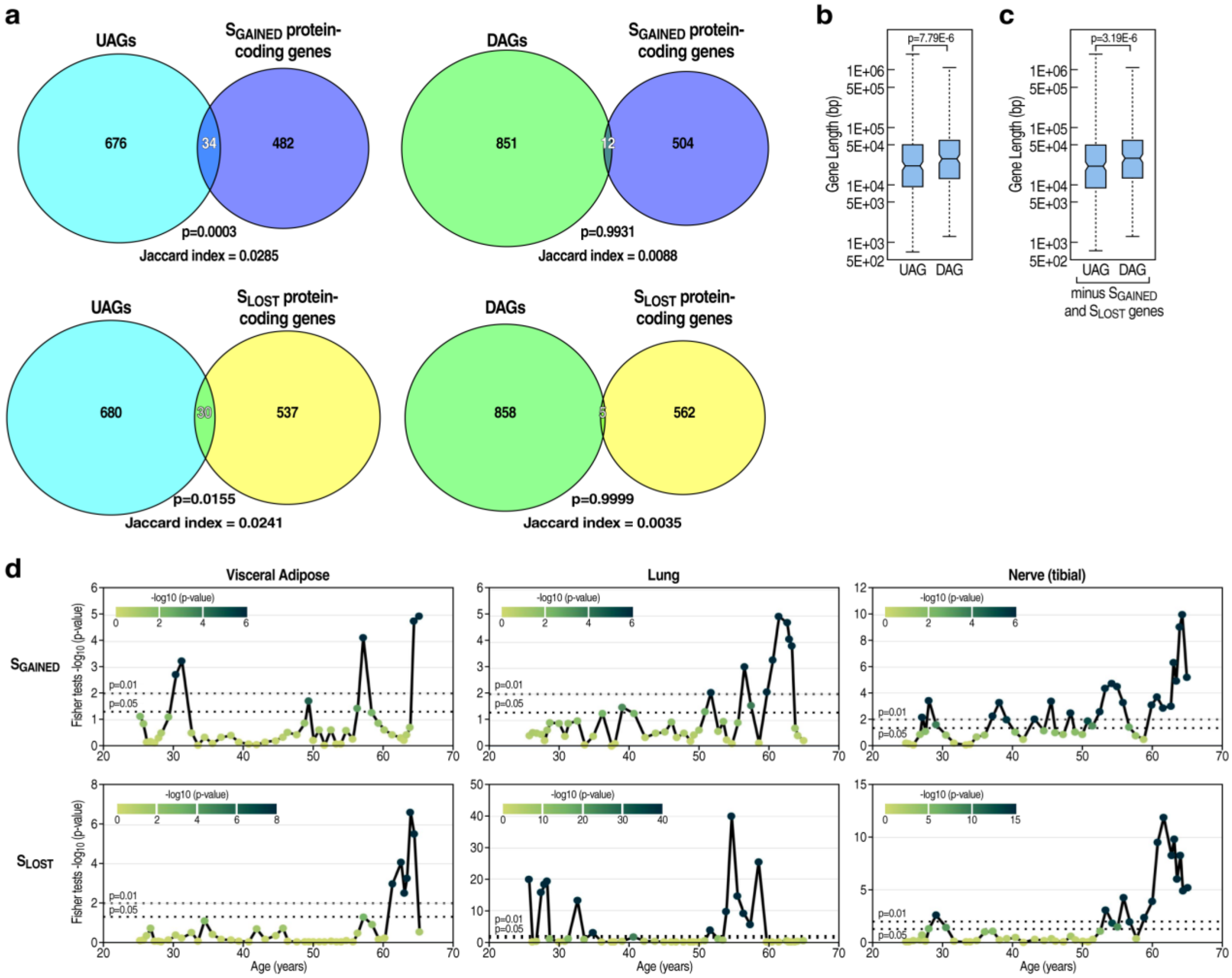

a
UAGs
SGAINED protein-coding genes
676
34
482
p=0.0003
Jaccard index = 0.0285
DAGs
SGAINED protein-coding genes
851
12
504
p=0.9931
Jaccard index = 0.0088
UAGs
SLOST protein-coding genes
680
30
537
p=0.0155
Jaccard index = 0.0241
DAGs
SLOST protein-coding genes
858
5
562
p=0.9999
Jaccard index = 0.0035
b
p=7.79E-6
Gene Length (bp)
UAG
DAG
c
p=3.19E-6
Gene Length (bp)
UAG
DAG
minus SGAINED and SLOST genes
d
Visceral Adipose
Lung
Nerve (tibial)
SGAINED
SLOST
-log10 (p-value)
Fisher tests -log10 (p-value)
p=0.01
p=0.05
Age (years)

Figure S3. Association of S_{GAINED} and S_{LOST} gene sets with tissue aging. (a) Overlap of S_{GAINED} and S_{LOST} gene sets with a collection of upregulated (UAGs) and downregulated (DAGs) age-associated genes (Jia et al. 2018). For consistency between lists, only protein-coding genes were considered in this analysis. *p* values are for statistical enrichment using Fisher's exact test. Jaccard index indicates similarity of compared lists. Area-proportional Venn diagrams were generated with BioVenn (Hulsen et al. 2008). (b, c) Box plots showing the distribution of gene lengths for UAGs and DAGs with overlapping S_{GAINED} and S_{LOST} genes either (b) included or (c) excluded. *p* values determined by two-sided Mann–Whitney *U* tests. (d) Enrichment analysis of S_{GAINED} and S_{LOST} gene sets in human aging DEGs based on GTEx samples from donors aged 20–70 years using the voyAGEr webtool (Schneider et al. 2024). The selected tissues were previously reported to diverge from the typical GLTD aging trend in GTEx transcriptomic data (Stoeger et al. 2022). See Table S2 for enrichment analysis performed on all available tissues.
